# Efficacy of omeprazole combined with endoscopic hemostasis in the treatment of patients with bleeding peptic ulcers

**DOI:** 10.1097/MD.0000000000047681

**Published:** 2026-02-28

**Authors:** Xiaozhu Fan, Huihui Cheng, Deyuan Lian, Yanling Miao, Jingjing Song, Shumin Niu

**Affiliations:** a Emergency and Critical Care Center, Hebei Engineering University Affiliated Hospital, Handan, Hebei, China; b Gastroenterology Department, Handan 285 Hospital of China Rongtong Medical and Health Group, Handan, Hebei, China.

**Keywords:** endoscopic hemostasis, omeprazole, peptic ulcer bleeding, proton pump inhibitor, rebleeding

## Abstract

Bleeding peptic ulcers remain a major cause of non-variceal upper gastrointestinal hemorrhage, and optimizing post-endoscopic pharmacologic therapy is essential to reducing early rebleeding and improving clinical outcomes. This study evaluated the therapeutic effectiveness of high-dose versus low-dose omeprazole following endoscopic hemostasis. This retrospective observational study included patients with endoscopically confirmed bleeding peptic ulcers treated between August 2022 and August 2025. Patients received either high-dose omeprazole (80-mg intravenous loading dose followed by continuous infusion at 4 mg/h) or low-dose omeprazole (40 mg intravenously once daily) after endoscopic hemostasis. Hemoglobin, hematocrit, rebleeding rates, clinical recovery parameters, and adverse events were collected. Participants were followed for 30 days to evaluate delayed rebleeding. Continuous variables were analyzed using independent-samples *t* tests, and categorical variables using chi-square tests. A total of 119 patients were included (high-dose: n = 58; low-dose: n = 61). High-dose omeprazole significantly reduced rebleeding at 72 hours (6.9% vs 21.3%, *P* = .025) and 30 days (10.3% vs 29.5%, *P* = .009). Greater increases in hemoglobin and hematocrit, lower transfusion volume, faster cessation of bleeding, earlier hemodynamic stabilization, and shorter hospitalization were also observed (all *P* < .001). Adverse event rates were comparable between groups. High-dose omeprazole combined with endoscopic hemostasis enhances hemostatic stability, accelerates clinical recovery, and reduces short-term rebleeding without increasing treatment-related adverse events.

## 1. Introduction

Peptic ulcer disease (PUD) remains one of the most common causes of non-variceal upper gastrointestinal bleeding (NVUGIB) worldwide. Despite advances in diagnostic and therapeutic strategies, ulcer-related hemorrhage continues to pose significant clinical and economic burden, particularly among older adults and individuals with multiple comorbidities. Peptic ulcer bleeding is associated with considerable morbidity, risk of recurrent hemorrhage, need for transfusion, prolonged hospitalization, and increased mortality.^[1,2]^ Although the global incidence of peptic ulcer disease has gradually decreased, the widespread use of antiplatelet agents, anticoagulants, and nonsteroidal anti-inflammatory drugs has sustained the prevalence of bleeding episodes, underscoring the importance of effective management approaches. Endoscopic hemostasis has become the cornerstone of treatment for high-risk bleeding ulcers. Techniques such as epinephrine injection, thermal coagulation, and mechanical clipping have demonstrated substantial efficacy in achieving immediate hemostasis.^[3,4]^ However, the risk of early rebleeding after technically successful endoscopic therapy remains clinically significant. Early rebleeding, typically occurring within the first 72 hours, is recognized as a major determinant of adverse outcomes. It frequently necessitates repeat endoscopy, blood transfusion, and, in severe cases, surgical or radiologic intervention. Therefore, optimizing adjunctive pharmacologic therapy after endoscopic treatment has become essential to improving clinical outcomes.

Acid suppression plays a critical role in stabilizing the post-hemostasis ulcer base. High intragastric acidity contributes to clot destabilization by impairing platelet aggregation and accelerating fibrinolysis. Proton pump inhibitors (PPIs) mitigate this process by maintaining the intragastric pH above the threshold required for stable clot formation. High-dose intravenous (IV) PPI therapy – typically involving an initial bolus followed by continuous infusion – has been recommended in contemporary guidelines for ulcers with high-risk stigmata after endoscopic therapy. Several randomized and observational studies have demonstrated reductions in rebleeding, transfusion requirements, and length of hospitalization with intensive PPI strategies.^[5,6]^ Nevertheless, variations in dosing regimens, population characteristics, and healthcare resource availability have led to ongoing debate regarding the optimal intensity of acid suppression. Omeprazole, one of the most widely used PPIs, offers predictable acid-suppressive effects and broad availability, making it particularly suitable for routine clinical use. While high-dose regimens have demonstrated efficacy in achieving early stabilization of intragastric pH, low-dose regimens continue to be used in many clinical settings due to concerns about cost, infusion requirements, or local practice patterns. Whether lower-intensity PPI therapy provides comparable protection against early and short-term rebleeding remains a matter of clinical interest, particularly in healthcare systems where continuous infusion protocols are not uniformly adopted.^[7,8]^

Given these considerations, evaluating the comparative therapeutic effects of different omeprazole dosing strategies following endoscopic hemostasis is essential for optimizing care in patients with bleeding peptic ulcers. Understanding the impact on rebleeding rates, hematologic recovery, clinical stabilization, and treatment-related safety can help refine evidence-based treatment protocols and inform the design of standardized management pathways for this common and potentially life-threatening condition.

## 2. Materials and methods

### 2.1. Study design

This study was approved by the Ethics Committee of Hebei Engineering University Affiliated Hospital. This retrospective observational study enrolled patients diagnosed with bleeding peptic ulcers who received treatment at our institution between August 2022 and August 2025. Patients were assigned to 2 treatment groups according to the therapeutic regimen administered during hospitalization. The observation group received high-dose omeprazole combined with endoscopic hemostasis, whereas the control group received low-dose omeprazole combined with endoscopic hemostasis. Inclusion criteria were: age ≥ 18 years; endoscopically confirmed peptic ulcer with active bleeding or recent stigmata of hemorrhage; availability of complete clinical, laboratory, endoscopic, and follow-up data; and receipt of omeprazole therapy and endoscopic hemostasis as the primary treatment. Exclusion criteria included: malignant gastric or duodenal lesions; recurrent bleeding secondary to varices, Mallory–Weiss tears, or other non-ulcer etiologies; prior gastric surgery or known anatomical abnormalities affecting ulcer healing; severe hepatic, renal, or cardiopulmonary dysfunction; concurrent use of anticoagulants that could not be discontinued; and pregnancy or lactation. The study was conducted in accordance with the ethical principles outlined in the Declaration of Helsinki and was approved by the institutional medical ethics committee. Written informed consent was obtained from all participants before data collection.

### 2.2. Treatment protocol

Observation Group (High-Dose Omeprazole + Endoscopic Hemostasis): Patients in the observation group underwent endoscopic hemostasis as the initial intervention. Hemostatic methods – including epinephrine injection, thermal coagulation, or hemoclip placement – were selected according to the Forrest classification and lesion characteristics. After endoscopic control of bleeding, patients received high-dose omeprazole consisting of an 80-mg IV loading dose followed by continuous IV infusion at 4 mg/h for 72 hours. After hemodynamic stabilization, therapy was transitioned to oral omeprazole 40 mg once daily for at least 4 weeks to facilitate ulcer healing and prevent rebleeding.

Control Group (Low-Dose Omeprazole + Endoscopic Hemostasis): Patients in the control group received identical endoscopic hemostasis procedures. Post-hemostasis acid suppression was administered as low-dose omeprazole 40 mg intravenously once daily for 72 hours. Subsequently, oral omeprazole 20 mg once daily was prescribed for 4 weeks as maintenance therapy.

Supportive measures, including fluid resuscitation, blood transfusion when indicated, discontinuation of ulcerogenic medications, and Helicobacter pylori eradication when appropriate, were provided according to routine clinical practice in both groups.

### 2.3. Data collection and follow-up

Clinical data were retrospectively extracted from the institutional electronic medical record system. Baseline variables included demographic characteristics (age, sex), vital signs at admission, comorbidities, medication history, Helicobacter pylori status, laboratory parameters (hemoglobin, hematocrit), and endoscopic findings such as ulcer location, size, and Forrest classification. Treatment-related information, including details of endoscopic hemostatic techniques and omeprazole dosing regimen, was also recorded.

Peri-interventional laboratory values were collected at predefined time points, including hemoglobin and hematocrit levels at baseline and at 24, 48, and 72 hours after treatment. Clinical recovery indicators – time to cessation of hematemesis or melena, time to hemodynamic stabilization, and length of hospital stay – were systematically documented. Safety outcomes were assessed through review of inpatient monitoring records for electrolyte disturbances, gastrointestinal symptoms, infusion-related reactions, and any serious adverse events requiring treatment modification or discontinuation. Hemodynamic stability was defined as the achievement of sustained systolic blood pressure ≥90 mm Hg without the need for ongoing vasopressor support, a heart rate ≤100 beats/min, and no clinical signs of ongoing active bleeding for at least 6 consecutive hours following initial resuscitation and endoscopic intervention.

All patients were followed for 30 days after discharge to evaluate the occurrence of delayed rebleeding. Follow-up was conducted through scheduled outpatient visits or standardized telephone interviews. Rebleeding was defined as recurrence of hematemesis or melena accompanied by hemodynamic instability, a drop in hemoglobin of ≥2 g/dL, or endoscopic confirmation of recurrent hemorrhage. Complete follow-up data were obtained for all enrolled patients.

### 2.4. Statistical analysis

All statistical analyses were conducted using IBM SPSS Statistics, version 28.0 (IBM Corp., Armonk). Continuous variables were assessed for normality prior to analysis and are presented as mean ± standard deviation. Between-group comparisons of continuous variables, including hemoglobin, hematocrit, clinical recovery times, and transfusion volume, were performed using independent-samples *t* tests. Categorical variables, such as rebleeding rates and adverse event frequencies, were expressed as counts and percentages, and analyzed using the chi-square test. All statistical tests were 2-sided, and a *P* value < .05 was considered to indicate statistical significance.

## 3. Results

### 3.1. Baseline characteristics

A total of 119 patients with bleeding peptic ulcers were included in the study, with 58 patients in the high-dose omeprazole group and 61 patients in the low-dose omeprazole group. The mean age was 57.6 ± 10.5 years in the high-dose group and 56.2 ± 11.0 years in the low-dose group (*t* = 0.709, *P* = .479). The proportion of male patients was 77.6% (45/58) and 70.5% (43/61), respectively (χ^2^ = 0.777, *P* = .378). Systolic blood pressure at admission was 105.0 ± 19.6 mm Hg in the high-dose group and 98.1 ± 22.0 mm Hg in the low-dose group (t = 1.803, *P* = .074). Baseline hemoglobin levels were 8.4 ± 1.2 g/dL and 8.1 ± 1.2 g/dL in the 2 groups, respectively (*t* = 1.363, *P* = .175). The incidence of shock on admission was 41.4% (24/58) in the high-dose group and 31.1% (19/61) in the low-dose group (χ^2^ = 1.349, *P* = .246). Nonsteroidal anti-inflammatory drug use was documented in 24.1% (14/58) and 24.6% (15/61) of patients, respectively (χ^2^ = 0.003, *P* = .954). Helicobacter pylori infection was positive in 67.2% (39/58) of the high-dose group and 55.7% (34/61) of the low-dose group (χ^2^ = 1.659, *P* = .198). Regarding ulcer location, gastric ulcers accounted for 48.3% (28/58) and 37.7% (23/61), and duodenal ulcers for 51.7% (30/58) and 62.3% (38/61) in the high-dose and low-dose groups, respectively (χ^2^ = 1.357, *P* = .244). The distribution of Forrest classification (Ia–Ib/IIa/IIb) was similar between the 2 groups (χ^2^ = 0.042, *P* = .979). The number of comorbidities (0, 1, or ≥2) also did not differ significantly between groups (χ^2^ = 0.429, *P* = .807). Overall, no statistically significant differences were observed in baseline characteristics, indicating that the 2 treatment groups were well balanced before intervention (Table 1).

**Table 1:** Baseline characteristics of patients in the 2 treatment groups.

Variable	High-dose omeprazole group (n = 58)	Low-dose omeprazole group (n = 61)	*t*/χ^2^	*P* value
Age, yr	57.6 ± 10.5	56.2 ± 11.0	0.709	.479
Male sex, n (%)	45 (77.6%)	43 (70.5%)	0.777	.378
Systolic blood pressure at admission, mm Hg	105.0 ± 19.6	98.1 ± 22.0	1.803	.074
Hemoglobin at admission, g/dL	8.4 ± 1.2	8.1 ± 1.2	1.363	.175
Shock on admission, n (%)	24 (41.4%)	19 (31.1%)	1.349	.246
NSAID use, n (%)	14 (24.1%)	15 (24.6%)	0.003	.954
Helicobacter pylori positive, n (%)	39 (67.2%)	34 (55.7%)	1.659	.198
Ulcer location, n (%)			1.357	.244
Gastric	28 (48.3%)	23 (37.7%)		
Duodenal	30 (51.7%)	38 (62.3%)		
Forrest classification, n (%)			0.042	.979
Ia–Ib	22 (37.9%)	23 (37.7%)		
IIa	20 (34.5%)	22 (36.1%)		
IIb	16 (27.6%)	16 (26.2%)		
Number of comorbidities, n (%)			0.429	.807
0	17 (29.3%)	18 (29.5%)		
1	19 (32.8%)	23 (37.7%)		
≥2	22 (37.9%)	20 (32.8%)		

NSAID = nonsteroidal anti-inflammatory drug.

### 3.2. Primary outcome: early and short-term rebleeding rates

Within the first 72 hours after endoscopic hemostasis, the incidence of rebleeding was lower in the high-dose omeprazole group than in the low-dose omeprazole group (4/58 [6.9%] vs 13/61 [21.3%]). The difference was statistically significant (χ^2^ = 5.045, *P* = .025). During the 30-day follow-up period, 6 of 58 patients (10.3%) in the high-dose group and 18 of 61 patients (29.5%) in the low-dose group experienced rebleeding, indicating a significantly reduced 30-day rebleeding rate in the high-dose omeprazole group (χ^2^ = 6.781, *P* = .009; Table 2).

**Table 2:** Comparison of rebleeding rates between the 2 groups.

Outcome	High-dose omeprazole group (n = 58)	Low-dose omeprazole group (n = 61)	χ^2^ value	*P* value
Rebleeding within 72 h	4 (6.9%)	13 (21.3%)	5.045	.025
Rebleeding within 30 d	6 (10.3%)	18 (29.5%)	6.781	.009

### 3.3. Hemodynamic and laboratory parameters

At baseline, hemoglobin and hematocrit levels were comparable between the 2 groups (hemoglobin: 8.4 ± 1.2 g/dL vs 8.1 ± 1.2 g/dL, t = 1.363, *P* = .173; hematocrit: 25.0 ± 3.5% vs 24.7 ± 3.6%, t = 0.461, *P* = .645). Following treatment, hemoglobin levels at 24, 48, and 72 hours after endoscopic hemostasis were consistently higher in the high-dose omeprazole group than in the low-dose group. At 24 hours, mean hemoglobin was 9.0 ± 1.1 g/dL versus 8.5 ± 1.2 g/dL (*t* = 2.371, *P* = .018); at 48 hours, 9.5 ± 1.1 g/dL versus 8.8 ± 1.2 g/dL (*t* = 3.319, *P* = .001); and at 72 hours, 9.9 ± 1.0 g/dL versus 9.0 ± 1.1 g/dL (*t* = 4.674, *P* < .001). A similar pattern was observed for hematocrit. At 24 hours, hematocrit was 27.0 ± 3.4% in the high-dose group and 25.5 ± 3.5% in the low-dose group (*t* = 2.371, *P* = .018); at 48 hours, 29.0 ± 3.3% versus 26.5 ± 3.4% (*t* = 4.070, *P* < .001); and at 72 hours, 30.0 ± 3.2% versus 27.0 ± 3.3% (*t* = 5.035, *P* < .001). The total transfusion volume during hospitalization was significantly lower in the high-dose omeprazole group than in the low-dose group (650 ± 220 mL vs 820 ± 260 mL, *t* = 3.857, *P* < .001), indicating a reduced requirement for blood transfusion in patients receiving high-dose omeprazole (Table 3).

**Table 3:** Comparison of hemoglobin, hematocrit, and transfusion volume between the 2 groups.

Parameter	High-dose omeprazole group (n = 58)	Low-dose omeprazole group (n = 61)	*t* value	*P* value
Hemoglobin (g/dL)				
Baseline	8.4 ± 1.2	8.1 ± 1.2	1.363	.173
24 h after treatment	9.0 ± 1.1	8.5 ± 1.2	2.371	.018
48 h after treatment	9.5 ± 1.1	8.8 ± 1.2	3.319	.001
72 h after treatment	9.9 ± 1.0	9.0 ± 1.1	4.674	<.001
Hematocrit (%)				
Baseline	25.0 ± 3.5	24.7 ± 3.6	0.461	.645
24 h after treatment	27.0 ± 3.4	25.5 ± 3.5	2.371	.018
48 h after treatment	29.0 ± 3.3	26.5 ± 3.4	4.070	<.001
72 h after treatment	30.0 ± 3.2	27.0 ± 3.3	5.035	<.001
Total transfusion volume (mL)	650 ± 220	820 ± 260	3.857	<.001

### 3.4. Clinical recovery parameters

The time to cessation of hematemesis or melena was significantly shorter in the high-dose group than in the low-dose group (18.0 ± 6.0 hours vs 24.0 ± 8.0 hours; *t* = 4.610, *P* < .001). Similarly, the time to hemodynamic stabilization was reduced in the high-dose group compared with the low-dose group (12.0 ± 4.0 hours vs 16.0 ± 5.0 hours; *t* = 4.804, *P* < .001). The length of hospital stay was also shorter in the high-dose group (7.0 ± 2.0 days vs 8.5 ± 2.5 days; *t* = 3.603, *P* < .001), indicating more rapid overall recovery in patients receiving high-dose omeprazole (Table 4).

**Table 4:** Comparison of clinical recovery parameters between the 2 groups.

Parameter	High-dose omeprazole group (n = 58)	Low-dose omeprazole group (n = 61)	*t* value	*P* value
Time to cessation of hematemesis/melena (h)	18.0 ± 6.0	24.0 ± 8.0	4.610	<.001
Time to hemodynamic stabilization (h)	12.0 ± 4.0	16.0 ± 5.0	4.804	<.001
Length of hospital stay (d)	7.0 ± 2.0	8.5 ± 2.5	3.603	<.001

### 3.5. Safety outcomes

The incidence of newly developed electrolyte abnormalities was 13.8% (8/58) in the high-dose omeprazole group and 11.5% (7/61) in the low-dose group, with no significant difference between groups (χ^2^ = 0.145, *P* = .703). Drug-related gastrointestinal symptoms occurred in 17.2% (10/58) and 14.8% (9/61) of patients, respectively (χ^2^ = 0.137, *P* = .711). Infusion-related reactions were reported in 5.2% (3/58) of patients in the high-dose group and 6.6% (4/61) in the low-dose group (χ^2^ = 0.103, *P* = .748). Serious adverse events requiring treatment modification or discontinuation were infrequent and showed no statistically significant difference between groups (3.4% [2/58] vs 4.9% [3/61]; χ^2^ = 0.160, *P* = .690). These findings indicate that high-dose omeprazole did not increase the risk of treatment-related adverse events compared with low-dose omeprazole (Table 5).

**Table 5:** Comparison of treatment-related adverse events between the 2 groups.

Safety outcome	High-dose omeprazole group (n = 58)	Low-dose omeprazole group (n = 61)	χ^2^ value	*P* value
Newly developed electrolyte abnormalities, n (%)	8 (13.8%)	7 (11.5%)	0.145	.703
Drug-related gastrointestinal symptoms, n (%)	10 (17.2%)	9 (14.8%)	0.137	.711
Infusion-related reactions, n (%)	3 (5.2%)	4 (6.6%)	0.103	.748
Serious adverse events requiring treatment modification or discontinuation, n (%)	2 (3.4%)	3 (4.9%)	0.160	.690

## 4. Discussion

The present study evaluated the efficacy and safety of high-dose IV omeprazole combined with endoscopic hemostasis in patients with bleeding peptic ulcers. Several consistent patterns were observed across early hemostatic outcomes, hematologic recovery, and clinical course. High-dose omeprazole significantly reduced both 72-hour and 30-day rebleeding rates compared with low-dose omeprazole. This reduction was accompanied by higher hemoglobin and hematocrit values during the first 72 hours, a lower cumulative transfusion requirement, shorter time to cessation of hematemesis or melena, earlier hemodynamic stabilization, and shorter length of hospital stay, without an increase in treatment-related adverse events. Collectively, these findings suggest that more intensive acid suppression in the immediate post-endoscopic period confers clinically meaningful benefits in NVUGIB due to peptic ulcers.

The observed reduction in rebleeding is biologically plausible. Sustained elevation of intragastric pH stabilizes platelet aggregation, reduces fibrin clot dissolution, and attenuates pepsin-mediated clot lysis. High-dose PPI regimens after successful endoscopic hemostasis have been shown to maintain intragastric pH above the threshold required for clot stability. Recent reviews of upper gastrointestinal bleeding management emphasize that high-dose IV PPI therapy, administered as a bolus followed by continuous infusion, is a central component of guideline-concordant care for high-risk peptic ulcers.^[9]^ In the present cohort, patients receiving high-dose omeprazole demonstrated more rapid hematologic recovery and a lower need for transfusion, which is consistent with improved control of ongoing or subclinical bleeding and more stable clot formation at the ulcer base. The parallel improvements in clinical recovery parameters support the view that the pharmacodynamic effects of intensive acid suppression translate into tangible patient-centered outcomes, including shorter hospitalization.^[10,11]^

Recent literature from the past 3 years provides important context for interpreting these results. Contemporary reviews of NVUGIB consistently reaffirm high-dose PPI therapy following endoscopic hemostasis as standard care for high-risk peptic ulcer bleeding, highlighting its role in reducing rebleeding, need for surgery, and mortality.^[12]^ These recommendations build on earlier randomized trials but are now supported by updated syntheses of real-world data and evolving practice patterns. More recently, the therapeutic landscape has expanded with the introduction of potassium-competitive acid blockers such as vonoprazan. A 2024 comparative study examining vonoprazan versus IV PPIs in patients with high-risk peptic ulcers reported that vonoprazan achieved non-inferior or superior control of rebleeding compared with conventional high-dose PPI therapy, underlining the continuing importance of potent acid suppression irrespective of the specific agent used.^[13,14]^ However, that study was conducted in centers with ready access to novel agents, and high-dose IV PPI remained the benchmark comparator. In contrast, the present study addresses a widely available and cost-effective regimen based on omeprazole, which is more feasible in many routine clinical settings.

Recent research has also explored PPI strategies in particularly high-risk subgroups. A 2024 observational study in patients undergoing transcatheter arterial embolization for refractory NVUGIB found that high-dose PPI regimens equivalent to ≥8 mg/h omeprazole were associated with favorable hemostatic outcomes in this critically ill population, reinforcing the principle that intensive acid suppression is beneficial when bleeding risk is high.^[15]^ Furthermore, a 2025 evidence-based review of PPI use across acid-related diseases emphasizes that while standard doses are sufficient for most indications, high-dose short-term regimens remain appropriate and evidence-supported for severe conditions such as peptic ulcer bleeding after endoscopic therapy.^[16]^ The present findings are broadly aligned with these contemporary data in showing that more intensive acid suppression improves early hemostatic outcomes. At the same time, this study differs by directly comparing a high-dose continuous infusion regimen with a lower-dose regimen within the same institutional context. While some earlier randomized trials suggested that lower or intermittent dosing might be non-inferior in selected patients, more recent guidelines and reviews tend to favor high-dose strategies in patients with high-risk stigmata after endoscopic therapy.^[9]^ The reduction in both 72-hour and 30-day rebleeding observed here is consistent with this guideline-oriented approach and supports the continued use of intensive PPI therapy in this clinical scenario. The biological benefits of sustained intragastric acid suppression may also help explain the more rapid clinical recovery observed in this study. By stabilizing clot formation and minimizing subclinical or recurrent bleeding, high-dose omeprazole likely facilitates earlier cessation of hematemesis or melena and promotes faster restoration of hemodynamic stability. These effects reduce ongoing blood loss and transfusion requirements, which in turn contribute to improved physiologic recovery and a shorter duration of hospitalization. Thus, the pharmacodynamic advantages of intensive PPI therapy appear to translate directly into clinically meaningful improvements in patient recovery and healthcare resource utilization.

From a clinical perspective, these results support the preferential use of high-dose IV omeprazole infusion following successful endoscopic hemostasis in patients with bleeding peptic ulcers at increased risk of rebleeding. The reduction in early and 30-day rebleeding, together with lower transfusion requirements and shorter hospital stays, indicates potential benefits not only for patient outcomes but also for resource utilization. Given that no significant increase in adverse events was observed, high-dose omeprazole appears to offer a favorable benefit–risk profile in routine practice, especially in institutions where newer acid-suppressive agents are not yet widely available. This study has several strengths. First, the comparison between high-dose and low-dose omeprazole was conducted in a relatively homogeneous cohort of patients with endoscopically confirmed bleeding peptic ulcers, all treated according to a standardized endoscopic protocol. Second, multiple clinically relevant endpoints were examined, including rebleeding at 2 time horizons, hematologic recovery, transfusion requirements, clinical recovery parameters, and safety outcomes. This multidimensional assessment provides a comprehensive view of the impact of dosing strategy on both hemostatic efficacy and patient trajectory. Third, the study reflects real-world practice in a contemporary setting, enhancing its external relevance for similar institutions.

However, several limitations should be acknowledged. The retrospective design introduces an inherent risk of selection bias and unmeasured confounding, despite comparable baseline characteristics between groups. Treatment allocation was not randomized, and subtle differences in clinical decision-making or supportive care may have influenced outcomes. The sample size, although adequate to detect differences in rebleeding and some secondary endpoints, may not be sufficient to identify rare adverse events or modest differences in safety outcomes. Additionally, the follow-up period was limited to 30 days; longer-term ulcer healing and recurrence were not systematically assessed. The study was conducted at a single center, which may limit generalizability to settings with different patient populations, endoscopic expertise, or supportive care protocols. Finally, intragastric pH was not directly monitored, so mechanistic inferences regarding acid suppression and clot stability are indirect, based on established pharmacologic principles and prior literature. Although baseline demographic, clinical, and endoscopic characteristics were well balanced between the high-dose and low-dose groups, treatment allocation was not randomized and may have been influenced by physician preference, perceived bleeding severity, or institutional practice patterns. Additionally, certain potential confounders – such as subtle differences in endoscopic technique, timing of intervention, or variations in supportive care – may not have been fully captured in the available clinical records. These factors could have influenced both hemostatic outcomes and recovery parameters and should be considered when interpreting the results.

## 5. Conclusions

High-dose omeprazole combined with endoscopic hemostasis significantly reduced early and 30-day rebleeding, improved hematologic recovery, shortened clinical stabilization time, and decreased hospital stay without increasing adverse events. These findings indicate that intensive acid suppression provides superior therapeutic benefit over low-dose therapy for bleeding peptic ulcers while maintaining an acceptable safety profile.

## Author contributions

**Conceptualization:** Xiaozhu Fan, Huihui Cheng, Deyuan Lian, Yanling Miao, Jingjing Song, Shumin Niu.

**Data curation:** Xiaozhu Fan, Huihui Cheng, Deyuan Lian, Yanling Miao, Jingjing Song, Shumin Niu.

**Formal analysis:** Xiaozhu Fan, Huihui Cheng, Deyuan Lian, Yanling Miao, Jingjing Song, Shumin Niu.

**Funding acquisition:** Xiaozhu Fan, Huihui Cheng, Shumin Niu.

**Investigation:** Shumin Niu.

**Writing – original draft:** Xiaozhu Fan, Shumin Niu.

**Writing – review & editing:** Xiaozhu Fan, Jingjing Song, Shumin Niu.
